# Health and aging in elderly farmers: the AMI cohort

**DOI:** 10.1186/1471-2458-12-558

**Published:** 2012-07-27

**Authors:** Karine Pérès, Fanny Matharan, Michèle Allard, Hélène Amieva, Isabelle Baldi, Pascale Barberger-Gateau, Valérie Bergua, Isabelle Bourdel-Marchasson, Cécile Delcourt, Alexandra Foubert-Samier, Annie Fourrier-Réglat, Maryse Gaimard, Sonia Laberon, Cécilia Maubaret, Virginie Postal, Chantal Chantal, Muriel Rainfray, Nicole Rascle, Jean-François Dartigues

**Affiliations:** 1 INSERM, ISPED, Centre INSERM U897-Epidémiologie-Biostatistique, Bordeaux, F-33000, France; 2 Univ. Bordeaux, Bordeaux, F-33000, France; 3 Institut de Neurosciences cognitives et intégratives d'Aquitaine, CNRS UMR 5287, Bordeaux, F-33076, France; 4 Ecole Pratique des Hautes Etudes, Bordeaux, F-33000, France; 5 Laboratoire Psychologie, Santé et Qualité de vie, EA 4139, Bordeaux, F-33076, France; 6 Pôle de Gérontologie Clinique, Centre Hospitalo-Universitaire de Bordeaux, Pessac, F-33604, France; 7 Résonnance Magnétique des Systèmes Biologiques, UMR 5536 CNRS, Bordeaux, F-33076, France; 8 INSERM, U567, Bordeaux, F-33076, France; 9 Centre Hospitalo-Universitaire CIC0005, Bordeaux, F-33000, France; 10 Centre Emile Durkheim UMR 5116, Bordeaux, F-33000, France

**Keywords:** Aging, Rural health, Agriculture, Cohort studies, Interdisciplinary studies

## Abstract

**Background:**

The health of the agricultural population has been previously explored, particularly in relation to the farming exposures and among professionally active individuals. However, few studies specifically focused on health and aging among elders retired from agriculture. Yet, this population faces the long-term effects of occupational exposures and multiple difficulties related to living and aging in rural area (limited access to shops, services, and practitioners). However, these difficulties may be counter-balanced by advantages related to healthier lifestyle, richer social support and better living environment. The general aim of the AMI cohort was to study health and aging in elderly farmers living in rural area through a multidisciplinary approach, with a main focus on dementia.

**Methods/design:**

The study initially included 1 002 participants, randomly selected from the Farmer Health Insurance rolls. Selection criteria were: being 65 years and older; living in rural area in Gironde (South-Western France); being retired from agriculture after at least 20 years of activity and being affiliated to the Health Insurance under own name. The study started in 2007, with two follow-up visits over 5 years. Baseline visits were conducted at home by a neuropsychologist then by a geriatrician for all cases suspected of dementia, Parkinson’s disease and depression (to confirm the diagnosis), and by a nurse for others. A large panel of data were collected through standardised questionnaires: complete neuropsychological assessment, material and social living environment, psychological transition to retirement, lifestyle (smoking, alcohol and diet), medications, disability in daily living, sensory impairments and some clinical measures (blood pressure, depression symptomatology, anxiety, visual test, anthropometry…). A blood sampling was performed with biological measurements and constitution of a biological bank, including DNA. Brain MRI were also performed on 316 of the participants. Finally, the three-year data on health-related reimbursements were extracted from the Health System database (medications, medical and paramedical consultations, biological examinations and medical devices), and the registered Long-Term Diseases (30 chronic diseases 100% covered by the Insurance System).

**Discussion:**

AMI is the first French longitudinal study on health and aging set up in a population of elderly farmers living in rural area through a multidisciplinary approach.

## Background

The health of farmers has been investigated, especially in relation to occupational exposures, such as toxic substances largely used in agriculture. Several studies reported that this population has a greater risk of several cancers (non-Hodgkin's lymphoma, Hodgkin's disease, multiple myeloma, prostate, connective tissue, skin, stomach, and brain) [1], respiratory diseases such as chronic obstructive pulmonary disease [2,3], musculoskeletal pain [4,5], reproductive outcomes [6] and accidents [7]. Some others also reported higher risk for neurologic diseases such as Parkinson’s disease [8,9] or cognitive decline and dementia [10-12]. On the other hand, some findings also suggested that this population would less often suffer from other conditions, such as cardiovascular diseases [1,5,6], some types of cancer (lung, colon, oesophagus and bladder [1][5]) or asthma (particularly for farm exposure in the childhood [13,14]). In terms of mortality, the literature suggests quite consistent results, showing a lower mortality for all causes of death, and particularly lower mortality by cancer [1,5]; only few studies suggested the reverse such as in Australia [15].

Most of the literature concerns individuals still professionally active and only few studies specifically focused on elderly former farmers. Yet, regarding the health specificities reported in the active population of farmers, some differences in terms of health and aging can be expected at long-term at older ages. In a study conducted on a sub-sample of the Paquid cohort on 1 507 elders, the relative risk of developing Alzheimer's disease for men occupationally exposed to pesticides was 2.4 (IC = 1.0-5.6), and even higher for Parkinson's disease with RR = 5.6 (CI = 1.5-21.6) [11]. This excess risk of Parkinson’s disease was also reported in a case–control study conducted on the Bordeaux area on 84 cases and 252 controls (OR = 2.2, CI = 1.1-4.3) [16]. Consequently, a specific study conducted in elderly former farmers may be relevant to better appreciate the long-term effects (delayed and/or cumulative effects) on health and aging of various agricultural exposures in the broad sense, such as to UV radiation, long-time hard work conditions, pesticides, high dust levels, diesel exhaust and solvents, endotoxins, animal virus.

Beyond the health effects of farming exposures, it appears also relevant to explore more largely the characteristics of the living environment of former farmers living in rural area, such as lower educational levels [17], lower retirement pensions in agriculture leading to rudimentary life conditions, geographical isolation with lack of public transportation and limited access to stores and services. Moreover, rural residents are more likely to face barriers in obtaining health care, with a growing desertification of medical and paramedical professionals (nurses, physical therapists, occupational therapists) [18]. The ongoing rationalization of health care provision may lead to potential consequences for rural people with longer travel times and waiting, lower levels of technology and more uneven resource distribution than in other areas [19].

However, these difficulties may be counter-balanced, at least in part, by some advantages of living in rural area. Compared to their urban peers, rural elders may have healthier lifestyle such as lower tobacco consumption [20,21], greater physical activity by pursuance of agricultural activities, gardening, walking, fishing, hunting (…) [22-24] and specific dietary habits possibly richer in fruits and vegetables, but the scarce literature on the topic in this specific population shows inconsistent results [25-27]. Moreover, they often benefit from well-developed social networks with better conviviality, more frequent social interactions and higher integration into social networks to provide informal social support and greater mutual aid [17,18]. In addition, living in rural area is often associated with lower stress and greater calmness, greater security, more spacious accommodations, often with green way.

Thus, the retirees from agriculture living in rural area may be particularly exposed to specific diseases, risk factors and difficulties in daily life, but may also benefit from protective factors which may significantly modulate the aging process. An extensive and global health approach could thus be particularly relevant in this population. Consequently, we decided to conduct the epidemiological AMI (Agrica-Msa-Institut fédératif de recherche en santé publique / Aging Multidisciplinary Investigation) cohort on health and aging in retired farmers living in rural area. Beyond the epidemiological approach, we paid from the very beginning of the research, a specific attention to the development of a multidisciplinary approach, involving epidemiologists, neurologists, neuropsychologists, psychologists, geriatricians, ophthalmologists, pharmaco-epidemiologists, demographists, sociologists, neuro-imagers, geneticists and biostatisticians. Through this global approach, the main objectives of AMI were (1) to describe social and environmental characteristics of the sample, (2) to study health and aging with a specific attention to neurodegenerative disorders like Alzheimer’s disease and related diseases and (3) to identify the agricultural and rural specificities which may impact health and aging. The present manuscript describes the rationale and the protocol of the AMI study.

## Methods/design

**Figure 1:**
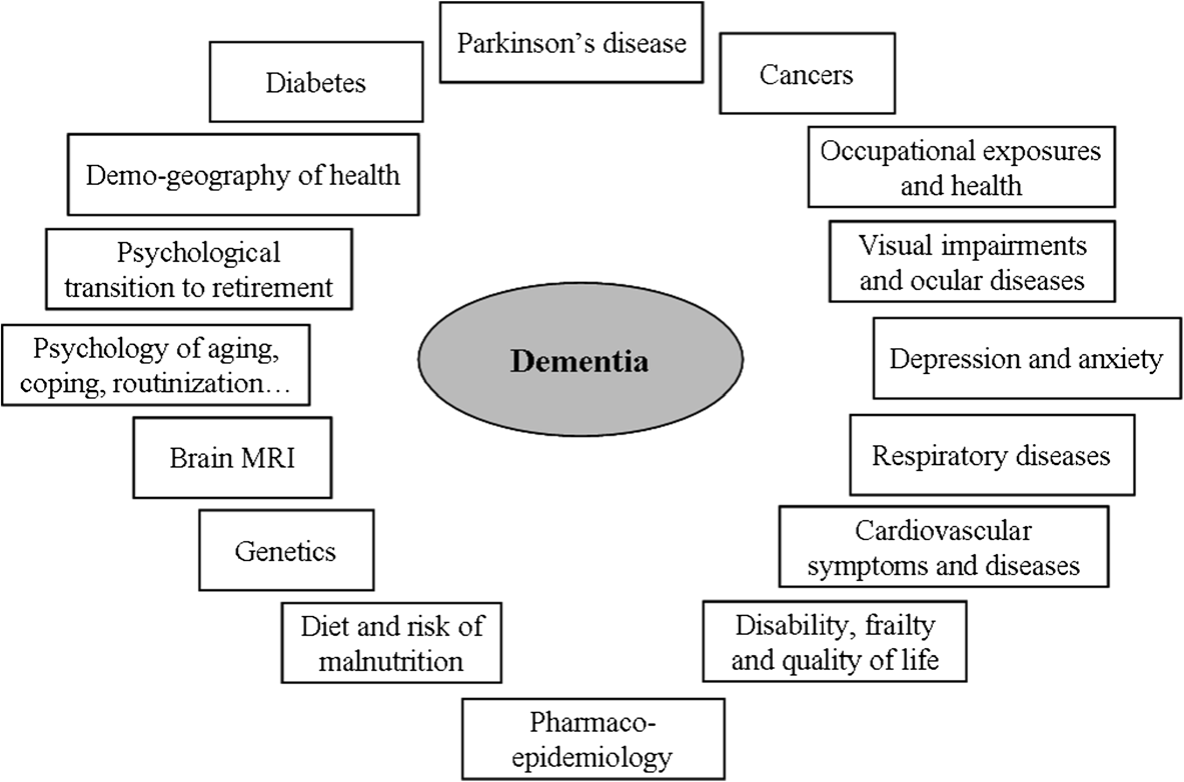
The different work packages developed in the AMI cohort.

The AMI cohort is an epidemiological prospective study on health and aging conducted in former farm-owners and farm-workers living in rural setting in South-Western France. Thanks to this collaborative approach, several work packages will be developed as presented Figure 1.

### Inclusion procedure

In 2007, the participants were randomly recruited from the reimbursement database of the unique French Farmer Health Insurance System (Mutualité Sociale Agricole, MSA) according to the following criteria: 1) Being aged 65 years and older at baseline, 2) Being retired from agriculture, 3) Having worked in agriculture for at least 20 years, 4) Being affiliated to the MSA under own name, and 5) Living in rural area (as defined by the French Institute of Statistics and Economic Studies, INSEE) in Gironde department, South-Western France. In order to get enough subjects from different socio-demographic profiles, we a priori determined the proportion of farm owners (one third of the sample) and of farm workers (two thirds). The Ethics Committee of the CHU (University Hospital) of Bordeaux approved this research according to the principles embodied in the Declaration of Helsinki.

### Visits and procedures at baseline

First, 2 193 individuals who fulfilled the inclusion criteria received a mail that briefly presented the study. Among them, 1 935 were contacted by telephone and for the 1 002 subjects who agreed to participate, the first visit was planned in the following days. Simultaneously, their GP’s were informed of this invitation and also received a brief presentation of the cohort.

### The neuropsychologist interview

The first interview was conducted at home by a specially trained neuropsychologist using a standardised questionnaire. Informed and written consent was obtained at the beginning of the visit for all participants. The following data were collected during a two-hour interview: age, gender, marital status, educational level, income, former professional activities and psychological transition to retirement (characteristics of the transition to retirement, retirement related affects [28], retirement related causalities [29], satisfaction and adaptation to occupational inactivity [30,31]). Social and material living environment was also investigated, such as social network, characteristics of the dwelling and accessibility of shops and services. The neuropsychologist also assessed restriction in activities of daily living through several scales, including basic Activities of Daily Living (ADL) [32], Instrumental ADL (IADL) [33], mobility [34], and homebound, as well as informal and professional assistance in daily difficulties. Regarding health-related data, the interviewer questioned the participants about subjective health, consultation to the GP’s and specialists (neurologist, geriatrician), and also collected all the medications currently used (the Anatomical Therapeutic Chemical ATC coding was used). Anxiety state was assessed using the State Trait Anxiety Inventory (STAI) [35], depressive symptomatology by the Center for Epidemiologic Studies-Depression scale (CES-D) [36] and the Mini-International Neuropsychiatric Interview (MINI) for major depressive episodes [37].

Finally, a complete battery of neuropsychological tests was administered by the neuropsychologist. Were explored: 1) subjective cognitive complaint according to the QPC scale (Questionnaire de Plainte Cognitive) [38]; 2) the Mini-Mental State Examination (MMSE), for global cognitive performance [39]; 3) the Free and Cued Selective Reminding Test RL/RI-16 items (FCSRT) [40] and 4) the story recall subtest of the Wechsler memory scale [41] for episodic memory; 5) the visual Delayed Matching-to-Sample task (DMS48) for visual recognition [42]; 6) the Goblet test (Mokri *et al.* submitted) for visuo-spatial working memory; 7) the Digit Symbol Substitution Task [43] for psychomotor speed; 8) the Wechsler Similarities test [43] for abstract thinking; and 9) the Isaacs Set Test [44] for verbal fluency. At the end of the visit, the neuropsychologist gave a clinical conclusion regarding possible dementia, Parkinson’s disease and depression.

### The health interview by a geriatrician or a nurse

Few weeks after the neuropsychological examination, a second home visit *(The health visit)* was performed in all participants, except for deceased, refusals and or other reasons. This visit was conducted by a nurse, except for individuals suspected of suffering from dementia, Parkinson’s disease and depression on the basis of the neuropsychologist’s examination. For these latter, the visit was conducted by a geriatrician, who could also conduct a clinical examination to confirm or infirm the diagnosis and specify the aetiology.

The health visit consisted in an evaluation of dietary habits using a short food frequency questionnaire, a detection of malnutrition by the Mini-Nutritional Assessment, [45] an assessment of alcohol and tobacco consumption and anthropometric measurements (weight, waist, calf, and upper arm circumferences, and self-reported height). Blood pressure was measured twice at different times of the visit using a validated digital electronic tensiometer (OMRON® 705IT). The Semmes-Weinstein monofilament was used to detect lower limb neuropathy or to identify "at risk" feet [46]. Muscular strength (using the JAMAR® dynamometer), timed 3-meter walking test, sitting down and waking up from a chair, one leg stance were also evaluated. Self-reported Parkinson’s disease and five symptoms were also examined (rest tremor, postural tremor, gait disturbance, micrographia and hypertonia). Respiratory symptoms were explored by a spirometry in order to detect obstructive dysfunction using Piko-6® and questionnaires on dyspnoea in daily activities and chronic obstructive pulmonary disease (using a validated questionnaire adapted from ECRHS study [47]). Finally, self-reported ocular diseases and visual impairment (self-reported and assessed using a reading test commonly used by French ophthalmologists (Parinaud test) or its equivalent for the illiterate (Rossano and Weiss test)), hearing impairment (deafness or self-reported difficulty following conversations in noisy situation), and dental problems and dentures use were also assessed. The homogeneity of the data collection between nurses and geriatricians was ensured by a similar training and a standardised questionnaire with detailed recommendations.

### The dementia diagnosis procedure

Finally, a case consensus conference attended by the geriatrician in charged of the *health visit* (LDC, SC) and three other dementia specialists’ clinicians (JFD, AFS, SA) was conducted to finally confirm or infirm the diagnosis. The aetiology was assigned according to the National Institute of Neurological and Communication Disorders and Stroke/Alzheimer’s Disease and Related Disorders Association (NINCDS-ADRDA) criteria [48] for Alzheimer’s disease, National Institute of Neurologic Disorders and Stroke/Association Internationale pour la Recherche et l’Enseignement en Neurosciences (NINCDS-AIREN) criteria for vascular dementia [49] and Diagnostic and Statistical Manual of Mental Disorders Third Edition Revided (DSM-III-R) [50] for Parkinson’s disease dementia.

### The self-administered questionnaire

Each participant was asked to fill in a self-administered questionnaire, which was picked up at the time of the *health visit*. This questionnaire concerned data on agricultural career, such as domains of activity, type of jobs and activity duration, pesticides exposures (using a question: “Have you ever been exposed to pesticides in your occupational activity?”) and also the level of complexity of professional activities (adapted from the work of Shooler *et al.*. [51]). We also collected data on characteristics and comfort of the housing and on familial and social support using the MOS Social Support Survey questionnaire [52]. Hobby practice at different period of life (at ages 18 years, 40 years and currently) was also collected using a questionnaires adapted in French from Wilson [53] and Schooler [54]. Finally, quality of life (using a specific scale for elders; scale in validation in the AMI cohort), proactive coping subscale [55] and routinization preferences were also assessed [56].

### Blood sampling

At a third step, a fasting blood sampling conducted at home has been proposed to all the participants. Biological measurements (blood glucose level after overnight fasting, glycosylated haemoglobin, creatinine blood level, cholesterol levels -total, HDL, LDL, LDL/HDL quotient, triglycerides, vitamins A and E) have been performed and a biological bank was constituted including DNA samples and plasma, serum and red blood cells samples kept frozen at −80°C.

### Three-year reimbursements of medical care by the Farmer Health Insurance System

Health reimbursements data were extracted from The Farmer Health Insurance database for the three years following the starting of the inclusions, i.e. from October 2007 to September 2010. In addition to the medications collected at the time of the neuropsychological visit, we thus obtained the three-year medication use (only those reimbursed by the Health Insurance, using the ATC codes). We also extracted data on medical (GP’s and specialists) and paramedical consultations (dentists, nurses, physical therapists, speech therapists, chiropodists…), biological examinations (blood examinations, imaging…), and reimbursed materials (optical correction, hearing and dental prosthesis, wheelchair, respiratory assistance devices…). Finally, we also obtained data on the Long-Term Diseases (“Affection Longue Durée”, ALD), which are the 30 chronic and costly diseases recognized by the French Insurance System and 100% covered.

### Neuro-imaging

**Figure 2:**
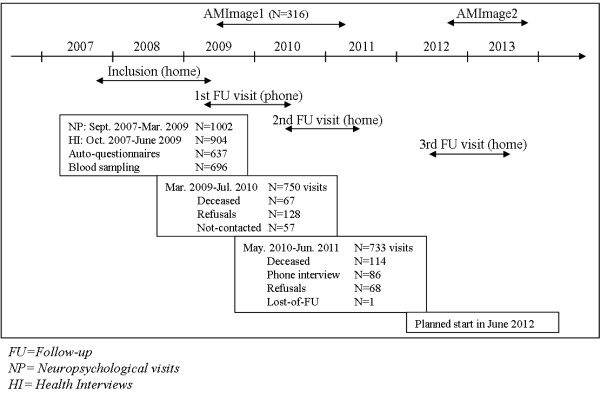
Flow chart of the longitudinal procedure of follow-up of the AMI cohort.

Brain MRI were performed on a sub-sample of the AMI cohort at the University Hospital of Bordeaux in the frame of the AMImage project in 2009–2011. A second wave of MRI will be conducted 3 years later in the frame of the AMImage2 project (Cf. Figure 2).

### Longitudinal follow-up

The participants included in 2007 will be seen at home three times between 2007 and 2013. After one year, a short phone interview is conducted by a neuropsychologist (Cf. Figure 2). All the other follow-up visits are conducted by a neuropsychologist but at home, following the same procedures than that of the baseline with: complete standardised neuropsychological testing, important modifications of living environment (house moving, bereavement…), health problems and hospitalization since the previous visit, medications, sensory and respiratory impairments, depressive symptomatology, disability, falls in the three last months, walking speed, diet and malnutrition risk, weight and height, tobacco and alcohol consumption. Similarly to baseline, an active research of dementia and Parkinson’s disease was conducted; all cases being confirmed or not by both a geriatrician (after a clinical examination at home) and the case consensus conference. We also used a self-administered questionnaire to collect longitudinal data on hobbies, quality of life, anxiety trait [35]and routinization preferences.

### Sample description and follow-ups

At baseline, the participation rate was 52%. As presented Figure 2, of the 1002 subjects of the initial sample, 904 had the health interview (N = 98, 73 refused, 16 were deceased and 9 were not visited for other reasons), of whom 204 received the visit of the physician for diagnosis confirmation. The self-administered questionnaire was filled-in by 637 of the participants, 696 subjects agreed having the blood sampling and 316 had brain MRI (Cf. Figure 2).

When comparing refusals and participants on the data available in the sampling dataset, no statistical differences were observed by age (mean 75.1 years (standard deviation, SD 6.6) vs. 75.0 years (6.6), p = 0.64), neither by gender (40.0% of women in the refusals vs. 37.5% in the participants, p = 0.27). We noticed a slight higher rate of refusal in the farm workers compared to the farm managers (50.2% and 45.4% respectively, p = 0.0531).

Compared to the general population of the area (census data of the Gironde area), the age distribution (21.6% of 65–69 years, 24.8 % of 70–74, 24.4% of 75–79 and 29.3% of 80 years and over) was not significantly different (respectively according to the census: 23.4%, 23.7%, 21.6% and 31.3%, p = 0.084). However, the AMI sample was significantly younger than the agricultural elderly population of former farmers of the area, since only 53.7% of our cohort were aged 75 years and over vs. 64.7% of the agricultural population (p < 0.0001). Moreover, the cohort was not representative in terms of sex, since participants had to be affiliated to the Health Insurance under own name to be selected from the initial dataset. The initial sample thus included 62.5% of men (compared to 40.4% in the general population and 45.8% in the agricultural population of the area).

As presented Figure 2, 733 subjects were visited at the first follow-up visit at home conducted 2 years later, 114 were deceased, 86 only accepted a phone interview, 68 refused the visit and 1 was lost of follow-up. The next visit will start in June 2012.

## Discussion

This is the first French longitudinal study on health and aging in a population exclusively constituted by elderly farmers living in rural area and conducted through an extensive multidisciplinary approach. Two characteristics of this population will be focused on: living in rural area with the associated difficulties but also its beneficial effects, and being retired from agriculture with the potential long-term effects of farming exposures and agriculture-related factors.

To develop a global and multidisciplinary approach, the initial protocol of research was constructed in collaboration with several research teams, involving a large panel of disciplines, ranging from epidemiology to social and human sciences, from neuro-imaging to genetics. Moreover, the Farmer Health Insurance datasets of reimbursements of drugs, consultations and services and the administrative record of Long-Term Diseases significantly enrich the database of the cohort, with a major interest in combination with the data actively collected over the follow-ups of the study. For instance, a combination of these data with the active research of dementia and its clinical diagnosis may be relevant to study the proportion of undiagnosed cases of dementia, the frequency of treated patients or to describe their medical and paramedical care pathway. Moreover, all the visits (neuropsychologist, nurse and geriatrician, blood sampling) were conducted at home to limit the selection bias, especially in a population of elderly people, potentially frail, disabled, living sometimes in very distant and isolated villages and without public transportation. For all the visits conducted out-of-home (such as for MRI), taxis were systematically used.

## Conclusion

The aging population and the expected related growing burden of chronic diseases and age-related disorders will increase demand for health and social services, especially for specialties focusing on elderly patients. In addition to the potential long-term effects of agricultural exposures, the elderly farmers living in rural area probably faces multiple difficulties in daily life, sometimes largely greater than their urban peers. Nevertheless, those difficulties might also be counterbalanced by advantages of living in the rural environment. The aging process being highly multifactorial, the global approach developed in this cohort, based on a large variety of scientific complementary disciplines is stimulating and promising.

## Competing interests

KP has no conflict of interest. FM has no conflict of interest. MA has no conflict of interest. IB has no conflict of interest. PBG has no conflict of interest concerning the content of this paper. VB has no conflict of interest. IBM has no conflict of interest concerning the content of this paper. CD has no conflict of interest concerning the content of this paper. AFR has no conflict of interest related to the publication of this manuscript. AFS has no conflict of interest. MG has no conflict of interest. SL has no conflict of interest. CM has no conflict of interest. VP has no conflict of interest. CR had no conflict of interest. MR has no conflict of interest concerning the content of the paper. NR has no conflict of interest. HA has no conflict of interest concerning the content of the paper. JFD has no conflict of interest concerning the content of the paper. Study sponsors played no role in the design and conduct of the study; collection, management, analysis, and interpretation of the data; and preparation, review, or approval of the manuscript.

## Authors' contributions

KP has made substantial contributions to conception and design and acquisition of data and interpretation of data; is the leader of the work package “Disability frailty and quality of life”; she has been involved in drafting the manuscript; and gave final approval of the version to be published. FM has made substantial contributions to the acquisition of data and to the statistical analyses; she has been involved in drafting the manuscript; and has given final approval of the version to be published. MA is the leader of the work package “Brain MRI” and is the PI of the AMImage projects. She has been involved in revising the manuscript critically for important intellectual content and gave final approval of the version published. HA has made substantial contributions to conception and design, and acquisition of data, supervising the neuropsychological aspects of the study; she has been involved in revising the manuscript critically for important intellectual content; and gave final approval of the version to be published. IB is the leader of the work package “Occupational exposures”; she has been involved in revising the manuscript critically for important intellectual content and gave final approval of the version published. PBG has contributed to the study conception and design; she is the leader of the work package “Diet and malnutrition”; she has participated in drafting the manuscript; and gave final approval of the version to be published. VB has made contributions to conception for some psychological data; she is one of the leaders of the work package “Psychology of aging”; she has been involved in revising the manuscript critically for important intellectual content; and gave final approval of the version to be published. IBM is the leader of the work package “Diabetes”; she has been involved in revising the manuscript critically for important intellectual content; and gave final approval of the version to be published. CD has made substantial contributions to conception and design; she is the leader of the work package “Visual impairments and ocular diseases”; she has been involved in revising the manuscript for important intellectual content; and gave final approval of the version to be published. AFR has participated to the pharmacoepidemiological conception of the AMI cohort and is the leader of the work package “Pharmacoepidemiology”; she has been involved in revising the manuscript for important intellectual content and gave final approval of the version to be published. AFS is the leader of the work package “Parkinson’s disease”; she has been involved in revising the manuscript critically for important intellectual content and gave final approval of the version published. MG is the leader of the work package “Demo-geography of health”; she has been involved in revising the manuscript critically for important intellectual content and gave final approval of the version published. SL has made contributions to conception for some psychological data; she is one of the leaders of the work package “Psychology of aging”; she has been involved in revising the manuscript critically for important intellectual content and gave final approval of the version published. CM is the leader of the work package “Genetics”; she has been involved in revising the manuscript critically for important intellectual content and gave final approval of the version published. VP has made contributions to conception for some psychological data; she is one of the leaders of the work package “Psychology of aging”; she has been involved in revising the manuscript critically for important intellectual content and gave final approval of the version published. CR participated in the design of the study, particularly for the respiratory section; she is one of the leaders of the work package “Occupational exposures”; she has been involved in revising the manuscript critically for important intellectual content and gave final approval of the version published. MR is responsible for the work package “Cancer”; she has been involved in revising the manuscript critically for important intellectual content and gave final approval of the version published. NR is one of the leaders of the work package “Psychology of aging”; she has been involved in revising the manuscript critically for important intellectual content and gave final approval of the version published. As PI, JFD has made substantial contributions to conception and design and interpretation of data and is also the leader of the work package “Dementia”; he has been involved in drafting the manuscript; and gave final approval of the version to be published.

## Authors' information

KP (PhD) is an epidemiologist and permanent researcher at the French Institute for Health and Medical Research (INSERM), at the INSERM Research Center U897 “Epidemiology and Biostatistics”, in the Team *Epidemiology and Neuropsychology of Cerebral Aging*, where she coordinates the axis research on functional aging and is responsible of the AMI cohort. She focused her researches on the relationships between cognition and activity restriction in daily living in population-based cohorts on cerebral and functional aging.

FM (MSc) is a statistician at the French Institute for Health and Medical Research (INSERM), in the Team *Epidemiology and Neuropsychology of Cerebral Aging*, where she is in charge of the statistical analyses of the AMI study and participates to the coordination of the study.

MA (MD, Ph D) is Hospital Practitioner and Professor at the University of Bordeaux, France. She is the associated leader of the team “Neuroimaging and human cognition” and affiliated with the lab “Cognitive and Integrative Neuroscience”. Her research interests are in the field of cognition, normal and pathologic, and neuroinflammation.

HA is a neuropsychologist (PhD) and permanent CNRS researcher in the Team “*Epidemiology and Neuropsychology of Cerebral Aging”* of the INSERM Unit 897 *“Epidemiology and Biostatistics”* where she coordinates the research axis on age-related cognitive decline.

IB is an epidemiologist and assistant professor in Occupational Health in the “Occupational and Environmental Health” team of the INSERM Center U897 and Bordeaux Hospital“. Her research program addresses the effects of pesticides in human populations, and especially the impact on cognitive functions, the risk of neuro-degenerative diseases, and the risk of brain tumors. She developed specific programs for pesticide exposure assessment in epidemiological programs.

PBG (MD, Ph D) is Professor of Epidemiology and Public Health at the University of Bordeaux, France. She is presently head of the team “Nutritional epidemiology” at the INSERM Research Center U897 “Epidemiology and Biostatistics”. Her main research field concerns epidemiology of aging with a particular interest in nutritional protective or risk factors of Alzheimer’s disease, based on the French PAQUID and Three-City cohort studies.

VB (Ph D) is a psychologist, associate professor at the Laboratory psychology, health, quality of life EA 4139, University Bordeaux Segalen. Her research is focused on routinization, psychological vulnerability and daily functioning in the elderly.

IBM (MD, PhD) is geriatrician, in the University Hospital of Bordeaux, professor of geriatric medicine at the University Bordeaux Segalen. She also develops research on the epidemiology of diabetes in the elderly and frailty in older people with diabetes.

CD is an epidemiologist (PhD) and permanent researcher at the INSERM Research Center U897 “Epidemiology and Biostatistics”, in the Team *Epidemiology of Nutrition*, where she coordinates the axis research on nutrition and eye diseases. She focused her researches on the relationships between nutrition, lifestyle and age-related eye diseases.

AFR (PharmD, PhD) is Associate Professor at the University of Bordeaux in France. Her research is focused on pharmacoepidemiology in the elderly and related to anticancer drugs in the post-licensing phase.

AFS is neurologist (MD, PhD student) in university hospital of Bordeaux, specialised in dementia and movement disorders and she is researcher in the Team *Epidemiology and Neuropsychology of Cerebral Aging* at the INSERM Research Center U897.

MG is a demographer and researcher at the Centre Emile Durkheim UMR 5116, University of Bordeaux Segalen. Her research interests are in the field of demography of health.

SL is a psychologist (PhD), associate professor of work and organizational psychology in the Laboratory Psychology, Health and Quality of life, EA 4139, University Bordeaux Segalen. Her research is focused on professional transitions and quality of life.

CM (Ph D) is post-doctoral fellow in genetics in the Team “*Epidemiology and Neuropsychology of Cerebral Aging”* of the INSERM Unit 897*“Epidemiology and Biostatistics”.* Her main research field concerns Telomere length, aging and dementia.

VP is a psychologist (PhD) associate professor at the Laboratory psychology, health, quality of life EA 4139, University Bordeaux Segalen. Her research is focused on cognitive processes, leisure and intellectual activities.

CR is chest physician, professor of pulmonology, responsible of medical unit in management of respiratory diseases and researcher at the French Institute for Health and Medical Research (Inserm), in the Team *Laboratory of Health, Working and Environment*, where she coordinates the axis research in the epidemiology of asthma and allergies in general population, with specific focus on the impact of environmental factors on respiratory health.

MR (MD) is geriatrician and professor at the University Bordeaux Segalen.

NR (PhD) is Professor of Health Psychology, in the Laboratory "psychology, health, quality of life" EA 4139, University Bordeaux Segalen. Her research is focused on two axes: life events, social resources and coping and the study of the consequences of stress at work on health.

JFD (MD, Ph D) is Professor of Epidemiology and Public Health at the University of Bordeaux, France. He is head of the team “Epidemiology and Neuropsychology of Aging” at the INSERM Research Center U897 “Epidemiology and Biostatistics”. His main research field concerns epidemiology of aging with a particular interest in nutritional protective or risk factors of Alzheimer’s disease, based on the French PAQUID and Three-City cohort studies. All authors read and approved the final manuscript.

## Pre-publication history

The pre-publication history for this paper can be accessed here:

http://www.biomedcentral.com/1471-2458/12/558/prepub
